# Indigo naturalis for inflammatory bowel disease: evidence from animal studies and molecular mechanisms

**DOI:** 10.3389/fphar.2025.1588233

**Published:** 2025-07-18

**Authors:** Jie Hu, Mengen Zhou, Li Huang, Xiutian Guo, Pingping Mei, Peng Li, Yiting Wang, Yan Chen

**Affiliations:** Department of Anorectal Surgery, Shanghai Municipal Hospital of Traditional Chinese Medicine, Affiliated to Shanghai University of Traditional Chinese Medicine, Shanghai, China

**Keywords:** indigo naturalis, inflammatory bowel disease, ulcerative colitis, Crohn’s disease, histopathological index

## Abstract

**Background:**

Indigo naturalis (IN) has been extensively used in prescriptions of traditional Chinese medicine to treat inflammatory bowel disease (IBD), particularly ulcerative colitis (UC). However, there is a lack of quantitative, evidence-based assessments from preclinical trials.

**Aims:**

Quantitative statistical evidence regarding the efficacy of IN in animal models of IBD remains insufficient. This study performed a meta-analysis to evaluate the therapeutic effects of IN in experiments with IBD.

**Methods:**

Relevant animal studies were identified from PubMed, Web of Science, Embase, China National Knowledge Infrastructure, SinoMed, and Wanfang databases. Two researchers independently conducted literature screening and risk-of-bias assessments using the CAMARADES 10-point quality checklist. Meta-analysis was performed using Review Manager 5.4, focusing on the histopathological index as the primary outcome measure.

**Results:**

Of the 15 eligible studies included, over half had low risks of bias in more than five items. Compared to controls, the histopathological index significantly improved after IN treatment (*n* = 151/137; SMD = −2.69 [-3.36, −2.02]; *p* < 0.00001). Subgroup analysis showed that a high dose of IN (>600 mg/kg; 4 studies, *n* = 31/22; SMD = −3.55 [-5.72, −1.39]; *p* < 0.001) was most effective in reducing the histopathological index. The IN group showed a significantly lower final disease activity index (DAI) score (*n* = 121/89; WMD = −1.69 [-2.18, −1.20]; *p* < 0.00001), greater percentage body weight recovery (*n* = 77/63; WMD = 9.99 [6.50, 13.49]; *p* < 0.00001), and longer colon lengths (*n* = 65/51; WMD = 0.95 [0.67, 1.24]; *p* < 0.00001) compared to controls. Additionally, IN treatment reduced IL-1β, IL-6, IL-8, and TNF-α expression while increasing IL-10 levels. These findings suggest that IN ameliorates inflammation by balancing innate and adaptive immunity, modulating the AhR/CYP1A1 signaling pathway, and altering gut microbiota structure.

**Conclusion:**

IN demonstrated significant therapeutic efficacy in preclinical models of IBD, particularly at dosages exceeding 600 mg/kg. It protected colonic mucosal integrity and exerted beneficial effects through multiple molecular pathways.

## Introduction

Inflammatory bowel disease (IBD) is a chronic disorder that affects the lower gastrointestinal tract and includes Crohn’s disease (CD) and ulcerative colitis (UC), with multiple unclear pathogenic factors (Gordon et al., 2023). Common symptoms of IBD patients include diarrhea, abdominal pain, weight loss, vomiting, and rectal bleeding (Prentice et al., 2022). The precise pathogenesis of IBD remains elusive. Extensive epidemiological studies have identified genetic predisposition, dysregulated immune responses, and environmental factors as contributors to IBD (Singh and Bernstein, 2022). In recent decades, IBD has become increasingly prevalent in developing regions such as Asia (including Asian immigrants in Western countries) and South America (Aniwan et al., 2022), making it a global public health concern. In Western countries, although IBD incidence has stabilized, its prevalence remains high at over 0.3%. Moreover, persistent IBD may lead to hyperplasia and neoplastic growth, posing additional public health challenges (Ng et al., 2017).

Current treatments for UC mainly include 5-aminosalicylic acid (5-ASA) steroids, immunosuppressants (6-methylmercaptopurine and azathioprine), and biological agents (Jeong et al., 2019). Treatment strategies for CD are similar but typically require longer immunosuppressive therapy (Dolinger et al., 2024). However, these treatments have drawbacks, including dependence, intolerance, loss of efficacy, drug resistance, and opportunistic infections, that present significant clinical challenges (Nakase, 2020). Consequently, satisfactory remission rates are not consistently achieved in clinical practice. Complementary and alternative therapies are therefore necessary for patients, clinicians, and researchers.

Indigo naturalis (IN) is an indigo-blue powder derived from the stems and leaves of the Acanthaceae plant *Baphicacanthus cusia* (Nees) Bremek., *Polygonaceae* plant *Polygonum tinctorium* Ait., or Cruciferous plant *Isatis indigotica* Fort. Its earliest medical documentation in China dates to the *Theory of Medicinal Properties* from the Tang Dynasty (627 A.D.). IN has been used for centuries to treat fever, hemoptysis, pediatric convulsions, oral ulcers, and sore throats (Yang et al., 2020). Its primary components are indirubin, indigo, and tryptanthrin (Naganuma, 2019). These components possess anticancer, anti-angiogenic, anti-inflammatory, and antimicrobial properties (Sun et al., 2021). IN has a long-standing application in treating inflammatory diseases, including psoriasis and IBD (Kakdiya et al., 2024; Zhang et al., 2022; Naganuma et al., 2018; Uchiyama et al., 2020; Yue et al., 2023). Clinical studies have indicated promising results of IN for UC treatment (Matsuno et al., 2022; Kudo et al., 2022; Saiki et al., 2021). However, only two randomized controlled trials have compared IN with a placebo. Further studies are required to clarify the efficacy and safety of IN (Kakdiya et al., 2024). Existing quantitative evidence is insufficient to draw definitive conclusions (Kim et al., 2024; Hu et al., 2024). Therefore, this study aimed to address these gaps by quantitatively analyzing previous research data, providing objective evidence to support the use of IN in IBD management.

## Materials and methods

### Literature search

Relevant animal studies were searched in PubMed, Web of Science, Embase, China National Knowledge Infrastructure, SinoMed, and Wanfang databases from inception to May 2025 without language restrictions. The following MeSH terms were used: “inflammatory bowel disease,” “IBD,” “colitis, ulcerative,” “ulcerative colitis,” “colitis,” “Crohn disease,” “Crohns disease,” “Crohn’s disease,” “Crohn’s enteritis,” “Indigo naturalis,” “Qing Dai,” “IN,” “Qing-dai,” and “indigo pulverata levis.” Abstracts and full texts of eligible studies were reviewed independently by two reviewers (Jie Hu and Li Huang). Any discrepancies were resolved through discussion with a third reviewer (Yan Chen).

### Eligibility criteria

#### Participants

There were no restrictions regarding animal strain, sex, or age. Animal models of IBD induced by dextran sodium sulfate (DSS), trinitrobenzene sulfonic acid (TNBS), oxazolone (OXZ), or ovalbumin-induced allergic enteritis were included. The intervention duration and frequency were without restrictions. Models involving radiation-induced colitis or interleukin-10 (IL-10)^−/−^ spontaneous chronic colitis were excluded.

### Interventions and comparisons

Animals in the treatment groups received IN without restrictions on dosage form, dose, administration route, or duration. Control groups received no treatment or vehicle administration. Studies without controls were excluded.

### Outcome measures

The primary outcome was the histological index, assessed by evaluating epithelial hyperplasia, loss of enterocytes, infiltration of lamina propria granulocytes, and monocyte infiltration using hematoxylin–eosin (HE) staining of colon tissue sections. Secondary outcomes included the disease activity index (DAI), final percentage change in body weight, and inflammatory cytokine levels (IL-1β, IL-10, IL-6, IL-8, and TNF-α).

### Study types

Animal studies evaluating the therapeutic effects of IN against IBD in rats and mice were included. *In vitro* studies and clinical case reports were excluded.

### Literature selection and data extraction

Two reviewers independently extracted data, which included first author, publication year, animal strain, weight and sex, number of animals, IBD induction method, IN administration details (dose, route, and timing), and outcome measures. Graphical data were extracted using GetData Graph Digitizer 2.24. Any disagreements were resolved by discussion with a third reviewer (Yan Chen).

### Assessment of risk of bias

Study quality was evaluated using the 10-point quality checklist from the Collaborative Approach to Meta-Analysis and Review of Animal Data from Experimental Studies (CAMARADES) (Bahor et al., 2021). Each criterion was rated as “yes,” “no,” or “unclear,” representing low, high, or unclear risk of bias, respectively. Two researchers (Li Huang and Xiutian Guo) independently completed the assessment. Discrepancies were resolved by consultation with a third reviewer (Yan Chen).

### Statistical analysis

Meta-analysis was conducted using RevMan 5.4 software. Outcomes measured in the same units were reported using mean difference (MD), and those in different units were presented as standardized mean difference (SMD). Heterogeneity was assessed using the Cochrane I^2^ statistic, where I^2^ > 50% indicated significant heterogeneity. A fixed-effects model was used when data consistency was acceptable; otherwise, a random-effects model was employed. Sensitivity analysis of primary outcome HI was then conducted to exclude one or more studies one-by-one to infer the reliability of the results.

Subgroup analyses were performed based on animal species, IBD modeling methods, IN administration timing, dose, and route. If ten studies or more were in the meta-analysis, funnel plots were used to test the potential risk of publication bias.

## Results

### Basic information of included studies

**FIGURE 1:**
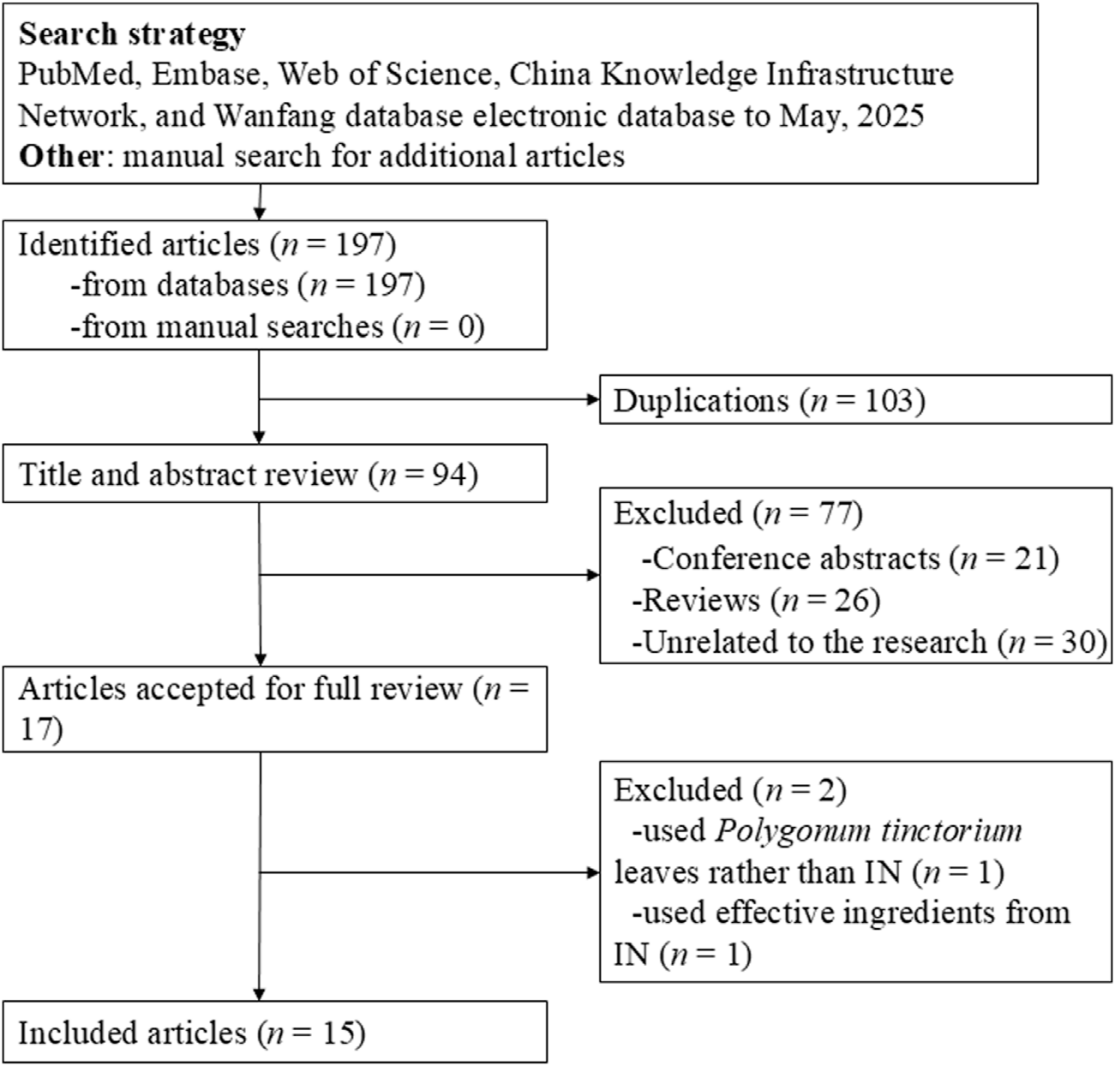
Summary of the literature identification and selection process.

Figure 1 presents the literature search and screening process. The initial search yielded 197 articles. After removing 103 duplicates, we further excluded 21 conference abstracts, 26 reviews, and 30 irrelevant records. Full-text review was conducted on 17 articles, of which 15 were ultimately included. Among the excluded articles, one utilized *Polygonum tinctorium* leaves instead of IN (Asari et al., 2022) and another used active ingredients extracted from IN (Xie et al., 2023). Included studies were written in English (n = 11) and Chinese (n = 4) (Hao et al., 2013; Liu et al., 2014; Adachi et al., 2017; Kawai et al., 2017; Wang et al., 2017; Liang et al., 2019; Xiao et al., 2019; Ozawa et al., 2020; Sun et al., 2020; Qiu, 2018; Yang et al., 2021; Fan et al., 2023; Yang et al., 2023; Zhang et al., 2023; Gu et al., 2024).

### Characteristics of included studies

Table 1 summarizes the characteristics of the included studies. Two studies employed rats, while the remainder utilized mice (Liu et al., 2014; Wang et al., 2017). For modeling methods, 13 studies used oral DSS, two used TNBS (Liu et al., 2014; Kawai et al., 2017), and one employed OXZ (Adachi et al., 2017). Modeling duration ranged from 5 to 12 days, except for two studies using a single-time induction method (Liu et al., 2014; Adachi et al., 2017). Sample sizes ranged from 14 to 60 animals. The dosage of IN ranged from 100 mg/kg to 1,680 mg/kg. Most studies applied intragastric administration, except two that mixed IN into powdered food (Adachi et al., 2017; Ozawa et al., 2020).

**TABLE 1:** Basic features of the studies included in the meta-analysis.

Study	Animals	Models	Groups	Sample size	Medication administration	Outcomes
Hao et al. (2013)	Male BABL/c mice, 7–8 weeks, weighing 24–29 g	5% DSS drinking water for 5 days	A. Control B. DSS C. DSS + IN 771.4 mg/kg D. DSS + Indirubin 9.64 mg/kg E. DSS + 5-ASA 292.86 mg/kg	10/10/10/10/10	IN dissolved in 0.5% CMC, with a gavage volume of 0.4 mL/g/d once a day for 7 days after drinking DSS solution for 6 days	HI, CD4^+^ T cells amount
Liu et al. (2014)	Male SD rats, weighing 200 ± 20 g	Inject 0.8 mL of 5% TNBS through rectum, complete within 1 min	A. Control B. TNBS C. TNBS + IN 1000 mg/kg D. TNBS + IN 600 mg/kg	5/5/5/5	Intragastric administration of IN once per day for 10 days after drinking DSS for 4 days	HE staining, IL-1β, IL-6, and IL-8
Adachi et al. (2017)	Male C57BL/6J mice, 6- to 8-week-old	100 μL of 0.5% OXZ in 50% ethanol intrarectally	A. Normal B. OXZ C. OXZ + IN 5% w/w	8/14/14	5% IN was mixed with powdered food from the day of colitis induction	HE staining, HI, body weight, endoscopic scores, IL-13, IL-4, TNF-α, and Siglec-F
Kawai et al. (2017)	Female C57BL/6J mice between 8 and 10 weeks	2% DSS drinking water for 7 days	A. DSS B. DSS + IN 120 mg/kg C. DSS + IN 600 mg/kg	7/7/7	Intragastric administration of IN once per day for 10 days, at the same time given drinking DSS	Weight loss, HI, colon length, HE staining, IL-10, Foxp3, IL-22, Cyp1A1, granzyme B, and CD4^+^ cells
		2% DSS drinking water for 7 days	A. DSS B. DSS + IN 600 mg/kg	7/7	Intragastric administration of IN once per day for 6 days after given drinking DSS	
		150 μL 2.5% TNBS by skin painting on day 1, 150 μL 2% TNBS intrarectally on day 8	A. TNBS B. TNBS + IN 300 mg/kg	7/7	Intragastric administration of IN once per day for 10 days, at the same time given drinking TNBS solution	
Wang et al. (2017)	Male SD rats (7 weeks, 180–220 g)	3% DSS drinking water for 7 days	A. Control B. DSS C. DSS + IN 4.2 g/kg D. DSS + IN 8.4 g/kg E. DSS + IN 16.8 g/kg F. DSS + SASP 400 mg/kg	8/8/8/8/8/8	Intragastric administration of IN once per day for 7 days, at the same time given drinking DSS solution	DAI, MPO, HI, HE staining, IL-1α, IL-1β, IL-18, EGF, and VEGF
Liang et al. (2019)	Male Kunming mice weighing 20 ± 2 g	3% DSS drinking water for 5–7 days	A. Control B. DSS C. DSS + SASP 125 mg/kg D. DSS + IN 100 mg/kg E. DSS + IN 200 mg/kg F. DSS + IN 400 mg/kg	6/6/6/6/6/6	Intragastric administration of IN twice a day for 7 days from the day of colitis induction	DAI, HI, IL-6, IL-10, IL-8, and TNF-α, structure of gut microbiota
Xiao et al. (2019)	7- to 8-week-old male C57BL/6 mice weighing 20–24 g	2% DSS drinking water for 5 days	A. Normal B. DSS C. DSS + IN 500 mg/kg/day D. DSS + IN 1000 g/kg/day E. DSS + SASP 0.20 g/kg/day	7/7/7/7/7	Intragastric administration of IN for 7 days after drinking DSS solution for 5 days	Body weight, DAI, colon length, HI, MPO, SOD, GSH-Px, CAT, IFN-γ, IL-17A/F, RORγt, TNF-α, IL-1β, Th1, Th17 and Treg differentiation, CD4^+^ cells, and p-STAT1
Ozawa et al. (2020)	5-week-old male C57BL/6JJmsSlc mice	1.0%–1.3% (w/v) DSS drinking water for 10 days	A. Normal B. DSS C. DSS +5.0% IN D. DSS +0.16% SASP E. DSS +0.16% 5-ASA	5/5/5/5/5	5% IN mixed with powdered food for 2.5 g/day, at the same time given drinking DSS solution	Body weight, survival rate, diarrhea score, bleeding score, HE staining, MMP3, IL-6, CXCL2, Ptgs2, Timp1, MMP9, Csf3, Lox, CCL7, CCL1, Hspb1, Ier 3, Spp1, IL-1β, IL-1r1
Sun et al. (2020)	Male SD rats, weighing 180–220 g)	4.5% (w/v) DSS drinking water for 7 days	A. Control B. DSS C. DSS + IN 600 mg/kg	8/8/8	Intragastric administration of IN once per day for 7 days, at the same time given drinking DSS solution	DAI, body weight, HI, HE staining, rectal bleeding, stool consistency, MPO, TGF-β, SCFAs, and GPRs
Qiu et (2018)	Male C57BL/6 mice weighing 20–24 g	5% DSS drinking water for 7 days	A. Control B. DSS C. DSS +5% IN 390 mg/kg	20/20/20	39 g/L IN 0.5% CMC with a gavage of 10 mL/kg once a day for 10 days from the day of colitis induction	DAI, HI, TNF-α, IL-6, TGF-β, IL-10, Bcl-2, Bax, and caspase-3
Yang et al. (2021)	Male BalB/c mice (20 ± 2 g)	3% DSS drinking water (w/v) for 7 days	A. Normal B. DSS C. DSS + IN 200 mg/kg D. DSS + Indigo 4.76 mg/kg E. DSS + Indirubin 0.78 mg/kg F. DSS + SSZ 200 mg/kg	6/6/6/6/6/6	Intragastric administration of IN for 7 days after drinking DSS solution for 3 days	DAI, body weight, colon length, HI, TDI, IL-1β, IL-6, TNF-α, MPO in serum and tissue, disturbed gut microbiota, TRL4/MyD88/NF-κB signaling pathway
Fan et al. (2023)	Male C57BL/6 mice (20–2 g) weighing 20–2 g	3% (w/v) DSS drinking water for 12 days	A. Normal B. DSS C. DSS + IN 200 mg/kg D. DSS + SASP 125 mg/kg	10/10/10/10	Intragastric administration of IN once per day for 12 days, at the same time given drinking DSS solution	DAI, HE staining, HI, TNF-a, IL-6, IL-8, IL-4, IL-10, and serum metabolic profiles
Yang et al. (2023)	C57BL/6 mice	4% DSS drinking water for 10 days	A. Normal B. DSS C. DSS + IN 600 mg/kg D. DSS + Indigo 300 mg/kg E. DSS + Indirubin 10 mg/kg F. DSS + Tryptanthrin 300 mg/kg	7/7/7/7/7	Intragastric administration of IN once per day for 7 days after drinking DSS solution for 4 days	Weight, colon length, HE staining, AHR, CYP1A1, IL-10, and IL-22
Zhang et al. (2023)	Male C57BL/6 mice weighing 20 ± 2 g	3% DSS drinking water for 5 days	A. Control B. DSS C. DSS + Qingcheng 520 mg/kg D. DSS + IN 400 mg/kg E. DSS + 5-ASA 200 mg/kg	10/10/10/10/10	IN dissolved in 0.5% CMC, with gavage 400 mg/kg once a day for 7 days from the day of colitis induction	DAI, IL-1β, IL-18, p-NF-κB, p65, GSDMD, and caspase-1
Gu et al. (2024)	Male C57BL/6 mice	Intragastric 3% DSS in distilled water for 10 days	A. Control B. DSS C. DSS + IN 600 mg/kg/day D. DSS + Indigo 300 mg/kg E. DSS + Indirubin 10 mg/kg F. DSS + Tryptophan 300 mg/kg/day	10/10/10/10/10/10	Intragastric administration of IN once per day for 10 days, at the same time given drinking DSS solution	Weight, colon length, DAI, HE staining, HI, the number of goblet cells, AHR, RORγt, CYP1A1, Foxp3, frequency ratios of Treg/Th17, IL-6, IL-10, IL-17 A and TGF-β

DSS, dextran sodium sulfate; IN, indigo naturalis; 5-ASA, 5-aminosalicylic acid; CMC, carboxymethylcellulose sodium; HI, histological index; SD, Sprague–Dawley; TNBS, trinitrobenzene sulfonic acid; HE, hematoxylin-eosin; IL-1β, interleukin-1β; IL-6, interleukin-6; IL-8, interleukin-8; OXZ, oxazolone; IL-13, interleukin-13; TNF-α, tumor necrosis factor alpha; SASP, sulfasalazine; DAI, disease activity index; IL-10, interleukin-10; MPO, myeloperoxidase; SOD, superoxide dismutase; GSH-Px, glutathione peroxidase; MMP3, matrix metalloproteinase-3; CXCL2, C-X-C motif chemokine 2; CSF3, colony-stimulating factor 3; CAT, catalase; IFN-γ, interferon-gamma; IL-17, interleukin-17; RORγt, retinoid-related orphan receptor gamma t; GSDMD, gasdermin D; TGF-β, transforming growth factor beta; Bcl-2, B cell lymphoma-2; AHR, aryl hydrocarbon receptor; CYP1A1, cytochrome P450 1A1; TGF-β, transforming growth factor beta.

### Assessment of risk of bias

Table 2 provides details on risk-of-bias assessments. The overall quality of the included studies was acceptable. Over half had a low risk of bias in five or more items. All studies except one (Qiu, 2018) underwent peer review. Four studies (Hao et al., 2013; Liu et al., 2014; Kawai et al., 2017; Yang et al., 2021) lacked statements of confirming ethical approval for animal use or compliance with animal welfare regulations and details of the experimental implementation. Nine studies reported temperature control during animal housing, and 11 described animal randomization. All studies except one (Liang et al., 2019), failed to report blinding during model establishment. Only four studies adopted blinding when evaluating outcomes. Twelve studies did not declare conflicts of interest. Additionally, no study reported the method for sample size calculation.

**TABLE 2:** Risk of bias summary.

Study	1	2	3	4	5	6	7	8	9	10
Hao et al. (2013)	+	?	+	?	?	—	+	?	?	+
Liu et al. (2014)	+	+	+	?	?	—	+	?	?	?
Adachi et al. (2017)	+	?	?	?	+	—	+	?	+	+
Kawai et al. (2017)	+	?	?	?	?	—	+	?	?	+
Liang et al. (2019)	+	+	+	+	+	—	+	?	+	+
Xiao et al. (2019)	+	+	+	?	+	—	+	?	+	+
Ozawa et al. (2020)	+	+	?	?	+	—	+	?	+	+
Sun et al. (2020)	+	+	+	?	+	—	+	?	+	+
Qiu et (2018)	-	?	+	?	+	—	+	?	+	?
Yang et al. (2021)	+	+	+	?	?	—	+	?	?	+
Fan et al. (2023)	+	?	+	?	?	—	+	?	+	+
Yang et al. (2023)	+	+	+	?	?	—	+	?	+	+
Zhang et al. (2023)	+	+	+	?	?	—	+	?	+	+
Gu et al. (2024)	+	+	+	?	?		+	?	+	+

(1) peer-reviewed journal; (2) temperature control; (3) animals randomly allocated; (4) blind established model; (5) blinded outcome assessment; (6) anesthetics used without marked intrinsic neuroprotective properties; (7) animal model (diabetic, advanced age or hypertensive); (8) calculation of sample size; (9) statement of compliance with animal welfare regulations; (10) possible conflicts of interest.

### Therapeutic effect of in IBD treatment

#### Histopathological indicators

**FIGURE 2:**
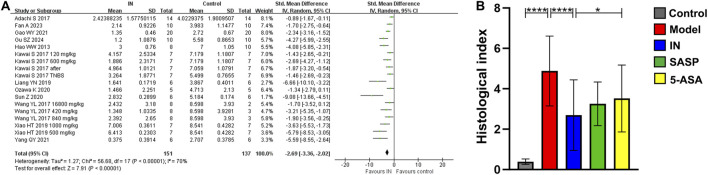
The meta-analysis result of histopathological index. **(A)**. The forest plot of histopathological index related to IN treatment compared with control; **(B)**. The bar chart of histopathological index of each group from meta-analysis.

Ten studies reported significantly reduced histopathological indices in the IN group (*n* = 151/137, SMD = −2.69 [-3.36, −2.02], *p* < 0.00001; Figure 2A), indicating protective effects on intestinal tissue. The effect was superior compared to the 3-ASA group (*p* < 0.05; Figure 2B). Although the heterogeneity in the included study could not be ignored, sensitivity analysis indicated that excluding individual studies does not affect the reliability of the results.

Subgroup analysis indicated that a higher IN dosage (>600 mg/kg, 4 studies, *n* = 31/22, SMD = −3.55 [-5.72, −1.39], *p* < 0.001; Table 3) produced the most significant reduction in histopathological indices compared with lower dosages (≤300 mg/kg, 4 studies, *n* = 29/29, SMD = −2.25 [-2.50, −1.99], *p* < 0.00001; 300–600 mg/kg, 8 studies, *n* = 72/67, SMD = −2.70 [-3.37, −2.03], *p* < 0.00001). No definitive conclusions were drawn regarding the influence of animal species, modeling methods, administration timing, or route of IN administration.

**TABLE 3:** Subgroup analysis of IN for histopathological index in IBD.

Outcomes	Studies	Sample size (IN/C)	SMD (95% CI)	*P* value
Animal species				
BALB/c mice	2	14/16	−3.13 [-4.76, −1.49]	0.0002
C57BL/6 mice	7	115/99	−2.18 [-2.67, −1.69]	<0.00001
Kunming mice	1	6/6	−2.23 [-2.58, −1.88]	<0.00001
SD rats	2	16/16	−3.32 [-5.31, −1.34]	0.001
Modeling method				
OXZ	1	14/14	−1.60 [-2.89, −0.31]	0.02
TNBS	1	7/7	−2.23 [-3.74, −0.73]	0.004
DSS<3%	3	40/40	−2.38 [-3.09, −1.68]	<0.0001
4%≥DSS≥3%	5	56/40	−2.95 [-3.78, −2.13]	<0.00001
DSS = 5%	3	34/36	−2.50 [-3.56, −1.44]	<0.00001
Administration time				
During modeling	9	102/86	−2.48 [-3.07, −1.89]	<0.00001
After modeling	4	49/51	−2.63 [-3.29, −1.98]	<0.00001
Dosage of IN				
≤300 mg/kg	4	29/29	−2.25 [-2.50, −1.99]	<0.00001
300–600 mg/kg	8	72/67	−2.70 [-3.37, −2.03]	<0.00001
>600 mg/kg	4	31/22	−3.55 [-5.72, −1.39]	0.001
Administration method of IN				
Intragastric administration	10	132/118	−2.56 [-2.99, −2.14]	<0.00001
Standard chow supplemented with IN	2	19/19	−1.97 [-3.32, −0.62]	0.004

IN, indigo naturalis; CI, confidence interval; IN/C, resveratrol/control; SMD, standard mean difference; OXZ, oxazolone; TNBS, 2,4,6-trinitrobenzenesulfonic acid; DSS, dextran sulfate sodium salt.

### Final DAI score, body weight change, and colon length

Among the 15 studies, nine used final DAI to assess the therapeutic efficacy of IN. The IN group showed significantly lower final DAI scores compared to controls (*n* = 121/89, WMD = −1.69 [-2.18, −1.20], *p* < 0.00001; Figure 2).

Nine studies assessed the final percentage change in body weight. The IN group demonstrated significantly improved body weight recovery compared to the model group (*n* = 77/63, WMD = 9.99 [6.50, 13.49], *p* < 0.00001; Table 4). Additionally, colon length was significantly greater in the IN group (*n* = 65/51, WMD = 0.95 [0.67, 1.24], *p* < 0.00001; Table 4).

**TABLE 4:** Body weight and inflammatory indicators of IN for IBD in animals.

Outcomes	Study	Sample size (IN/C)	WMD/SMD (95% CI)	*P* value
DAI score	9	121/89	−1.69 [-2.18, −1.20]	<0.00001
Body weight	9	77/63	9.99 [6.50, 13.49]	<0.00001
Colon length	7	65/51	0.95 [0.67, 1.24]	<0.00001
Inflammatory indicators				
TNF-α	6	68/56	−6.43 [-9.43, −3.42]	<0.0001
IL-8	4	48/48	−5.55 [-7.75, −3.35]	<0.00001
IL-6	7	81/63	−4.41 [-6.55, −2.16]	0.0003
IL-10	6	72/60	7.67 [4.07, 11.26]	0.0001
IL-1β	5	72/38	−5.53 [-7.51, −3.55]	<0.0001

IN/C, indigo naturalis/control; SMD, standard mean difference; WMD, weighted mean difference; CI, confidence interval; TNF-α, tumor necrosis factor-α; IL-8, interleukin-8; IL-6, interleukin-6; IL-10, interleukin-10; IL-1β, interleukin-1β.

### Effects of in on inflammatory indicators

Levels of IL-1β (five studies, n = 72/38, SMD = −5.53 [-7.51, −3.55], p < 0.0001), TNF-α (six studies, *n* = 68/56, SMD = −6.43 [-9.43, −3.42], *p* < 0.0001), IL-6 (seven studies, *n* = 81/63, SMD = −4.41 [-6.55, −2.16], *p* = 0.0003), and IL-8 (four studies, *n* = 48/48, SMD = −5.55 [-7.75, −3.35], *p* < 0.00001) significantly decreased following IN treatment. Conversely, IL-10 levels increased significantly (six studies, *n* = 72/60, SMD = 7.67 [4.07, 11.26], *p* = 0.0001). Detailed data are shown in Table 5.

**TABLE 5:** Risk-of-bias summary.

Study	Proposed mechanism	Specific direction
Hao et al. (2013)	Inhibited inflammation	Reduced CD4 T cell expression in mouse spleen.
Liu et al. (2014)	Inhibited inflammation	Reduced IL-1β, IL-6, IL-8 and TNF-α.
Adachi et al. (2017)	Inhibited inflammation	Dramatically altered gut flora and no longer exacerbated colitis when colitis was induced after gut flora depletion.
Kawai et al. (2017)	Inhibited inflammation	Ameliorated colitis through AhR signaling activation, increased the expression of anti-inflammatory cytokines, and resulted in the expansion of IL-10-producing CD4^+^ T cells and IL-22-producing CD3^−^RORγt^+^ cells, but not CD4^+^Foxp3^+^ regulatory T cells.
Wang et al. (2017)	Inhibited inflammation and increased colonic mucosal damage repair	Reduced the expression of inflammatory cytokines and increased the expression of colonic mucosal repair-related cytokines and occludin protein.
Liang et al. (2019)	Inhibited inflammation and modulated the balance of gut microbiota	Downregulated pro-inflammatory cytokines and up-regulating anti-inflammatory cytokines, downregulated the relative quantity of *Turicibacter* and up-regulated the relative quantity of *Peptococcus*.
Xiao et al. (2019)	Suppressed colonic oxidative stress and restraining colonic Th1/Th17 responses	Activated AMPK/Nrf-2 signals and inhibited STAT1/STAT3 signals, p-STAT1 and p-STAT3.
Ozawa et al. (2020)	Inhibited inflammation, and inhibited bleeding	Suppressed IL-1β-induced NO production.
Sun et al. (2020)	Inhibited inflammation and regulated microbiota-butyrate axis	Alleviated inflammation through a mechanism of the microbiota-butyrate axis, particularly alterations in *Ruminococcus_1* and *Butyricicoccus* abundances.
Qiu et (2018)	Inhibited inflammation and inhibited excessive cell death	Regulated inflammatory mediators, such as TNF-α, IL-6, TGF-β, and IL-10, inhibited excessive cell death by affecting apoptosis factors Bcl-2, Bax, caspase-3
Yang et al. (2021)	Inhibited inflammation and adjusted gut microbiota structure	Reduced IL-1β, IL-6, and TNF-α by inhibiting TLR4/MyD88/NF-κB signal transduction, reduced IgA and IgG both in serum and colon tissue, and adjusted gut microbiota structure by reducing the ratio of Firmicutes/Bacteroidetes and increasing the abundance of probiotics.
Fan et al. (2023)	Inhibited inflammation, and modulated metabolism	Modulated primary bile acid biosynthesis, AA, as well as the metabolism of unsaturated fatty acid.
Yang et al. (2023)	Inhibited inflammation	Regulated the expressions of inflammatory factors IL-10 and IL-22 by activating the AhR/CYP1A1 signaling pathway.
Zhang et al. (2023)	Inhibited pyroptosis	Inhibited pyroptosis by regulating NF-κB signaling.
Gu et al. (2024)	Inhibited inflammation and promoted mucosal healing	Modulated pro-inflammatory (IL-6 and IL-17 A) and anti-inflammatory (IL-10 and TGF-β1) factors and Treg/Th17 cell ratios, promoted mucosal healing and improved UC symptoms, potentially through AHR-Th17/Treg pathway regulation

IL-1β, interleukin-1β; IL-6, interleukin-6; IL-8, interleukin-8; TNF-α, tumor necrosis factor alpha; AhR, aryl hydrocarbon receptor; IL-22, interleukin-22; RORγ, RAR-related orphan receptor gamma; AMPK, AMP-activated protein kinase; Nrf-2, nuclear factor E2-related factor; STAT1, signal transducer and activator of transcription 1; STAT3, signal transducer and activator of transcription 3; NO, nitric oxide; TGF-β, transforming growth factor beta; IL-10, interleukin-10; bcl-2, B cell lymphoma-2; Bax, Bcl-2, associated X; TLR4, toll-like receptor 4; MyD88, myeloid differentiation primary response 88; NF-κB, nuclear factor-kappaB; CYP1A1, cytochrome P450 1A1.

### Publication bias

**FIGURE 3:**
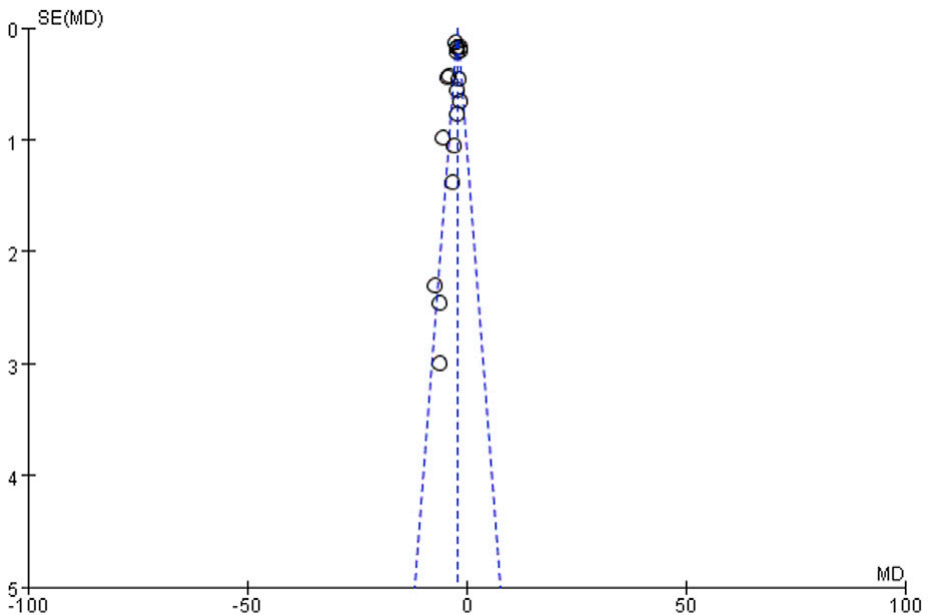
Funnel plot based on histopathological index.

Funnel plot analysis of histopathological indices demonstrated significant asymmetry, suggesting potential publication bias (Figure 3).

## Discussion

### Main results

This study is the first quantitative meta-analysis to assess the efficacy of IN for IBD treatment using animal models. The overall quality of the 15 included studies was acceptable. The results indicated that IN effectively reduced IBD severity, preserved colon length, and maintained body weight by inhibiting intestinal inflammation. High-dose IN (>600 mg/kg) was more effective than low (≤300 mg/kg) and medium (300–600 mg/kg) doses in reducing the HI.

### IN inhibited inflammation via regulating T cell differentiation

**FIGURE 4:**
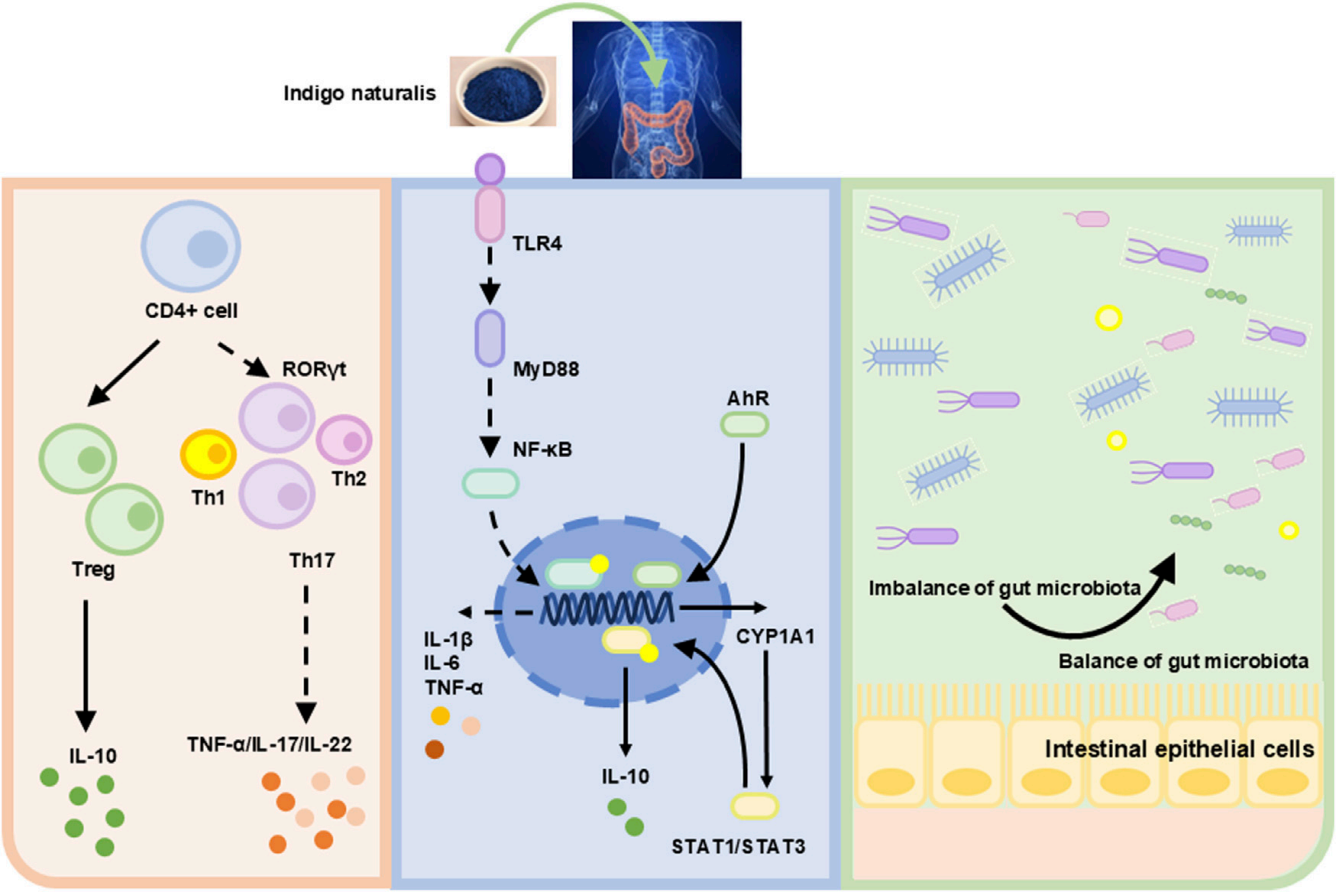
Mechanism diagram of IN alleviating IBD.

Cytokines mediate activated T-cell differentiation (Luckheeram et al., 2012). The pro-inflammatory cytokines IL-12 and IL-4 induce T cell differentiation into Th1 and Th2 cells, respectively (Ruterbusch et al., 2020). Retinoic acid receptor-related orphan receptor γ (RORγ) activates Th17 cells. Other factors, such as IL-16, also influence cell differentiation (Wu and Wan, 2020). The transcription factor forkhead box P3 (FOXP3) in regulatory T cells (Tregs) is activated by TGF-β. Elevated levels of IL-10 and TGF-β promote anti-inflammatory responses (Yue et al., 2024). RORγt, a transcription factor in innate lymphoid cells and Th17 cells, is upregulated by Tregs, specifically inhibiting type-17 immune reactions in colonic mucosa (Ren and Li, 2017). This regulatory function of Tregs exerts anti-inflammatory effects, contributing to IBD remission. Research showed that IN promoted the expansion of IL-10-producing CD4^+^ T cells and IL-22-inducing CD3^−^RORγt^+^ cells but did not significantly influence CD4^+^Foxp3^+^ Tregs (Kawai et al., 2017) (Figure 4).

### IN inhibited inflammation through regulating the AhR/CYP1A1/STAT pathway and TLR4/Myd88 signaling pathway

The immune system consists of innate and adaptive immunity. Innate immune cells include dendritic cells, macrophages, and neutrophils, which recognize microbial patterns or products (Bruner et al., 2023). Adaptive immune cells, including B and T cells, recognize specific antigens. A balanced interaction between innate and adaptive immunity prevents excessive immune responses to commensal microbiota (Kelsen and Sullivan, 2017). Disruption of this balance leads to intestinal inflammation.

Studies have shown that microbe-associated molecular patterns (MAMPs) interacted with toll-like receptors (TLRs) or other mucosal pattern recognition receptors (PRRs) in innate immunity, potentially alleviating IBD symptoms through probiotic-associated effects (Esmaealzadeh et al., 2024). TLR4 is a primary sensor for lipopolysaccharide (LPS) derived from Gram-negative bacteria. Research indicated that TLR4 overexpression aggravated inflammation and intestinal injury in DSS-induced colitis models. Conversely, TLR4 knockout provided protection (Chen et al., 2019; Fukata et al., 2009). Thus, TLR4 signaling may significantly influence intestinal damage and repair. IN inhibits innate immunity by regulating the TLR4/MyD88/NF-κB signaling pathway (Yang et al., 2021) (Figure 4).

The aryl hydrocarbon receptor (AhR) consists of 805 amino acids. Upon activation, AhR dissociates from a protein complex and binds as a dimer to the AhR nuclear translocation protein in the nucleus, promoting downstream gene transcription (Marafini et al., 2024). Research has demonstrated reduced AhR mRNA expression in patients with UC (Monteleone et al., 2011). Animal experiments indicated that colon inflammation in UC mice worsened after treatment with AhR antagonists. AhR knockout UC mice displayed varying degrees of colon damage, including crypt deformation, epithelial cell necrosis, oedema, and accumulation of leukocytes and neutrophils (Arsenescu et al., 2011). This suggests that upregulation of AhR expression could mitigate colonic inflammation. Activated AhR can upregulate cytochrome P450 1A1 (CYP1A1) expression. Activation of the AHR/CYP1A1 signaling pathway enhances IL-10 expression and secretion, consequently increasing IL-10 receptor (IL-10R1) expression in intestinal epithelial cells (Lanis et al., 2017). IL-10 binds to IL-10R1, inducing phosphorylation of the signal transducer and activator of transcription 3 (STAT3). Phosphorylated STAT3 promotes intestinal mucosal healing by increasing the transcription of anti-apoptotic and proliferation-related genes. Clinical studies revealed lower IL-10 expression in the colonic mucosa of moderate to severe UC patients compared to mild UC or normal controls (Wittmann Dayagi et al., 2021). Additionally, IL-10 knockout mice spontaneously develop enteritis (Geng et al., 2021). This review indicated that IN alleviates colitis by activating AhR signaling and inhibiting STAT1/STAT3 signals and IL-10 (Kawai et al., 2017; Xiao et al., 2019; Yang et al., 2023).

### IN modulated gut microbiota structure

Maintaining intestinal homeostasis requires the close interplay of immune, environmental, and genetic factors, as well as commensal microbiota. An imbalance in gut microbiota, especially involving pathogenic bacteria, disrupts intestinal mucosal immunity and causes mucosal damage. This increases intestinal permeability, allowing microbial invasion and ultimately triggering dysregulated mucosal immune responses (Franzosa et al., 2019). Previous studies have revealed altered gut microbiota composition in UC patients, characterized by reduced microbial diversity and decreased Firmicutes abundance (Vestergaard et al., 2024). Gut metabolites, derived from diet and microbiota-produced compounds, affect human metabolism. Short-chain fatty acids (SCFAs), including propionate, butyrate, and acetate, are produced by gut bacteria during dietary fiber decomposition. SCFAs regulate histone deacetylase activity, gene expression, cell proliferation, and immune responses. Butyrate specifically protects against colitis by promoting Treg cell differentiation and enhancing macrophage antibacterial activity. Studies reported reduced stool butyrate levels and decreased butyrate-producing bacteria in IBD patients (Franzosa et al., 2019). IN significantly altered gut microbiota, and microbiota depletion exacerbated colitis severity following induction (Adachi et al., 2017). IN downregulated the abundance of *Turicibacter* and upregulated *Peptococcus*. Another study demonstrated that IN decreased the Firmicutes*/*Bacteroidetes ratio and enhanced probiotic abundance, reshaping the gut microbiota composition (Yang et al., 2021; Liang et al., 2019). Furthermore, IN attenuated inflammation via the microbiota-butyrate axis, particularly by increasing the abundance of *Ruminococcus 1* and *Butyricicoccus*. Additionally, IN modulated primary bile acid biosynthesis, arachidonic acid (AA) metabolism, and unsaturated fatty acid metabolism (Fan et al., 2023; Sun et al., 2020).

### Dose of IN for IBD

This study found that IN doses exceeding 600 mg/kg demonstrated significant therapeutic efficacy in treating IBD, particularly in reducing HI. Among the included studies, four explored the relationship between dosage and efficacy. Two studies administered IN at 600 mg/kg and 1,000 mg/kg, finding that 1,000 mg/kg/day was superior in increasing rat body weight, improving DAI, and reducing serum IL-1β, IL-6, and IL-8 levels (Liu et al., 2014; Xiao et al., 2019). Another study compared doses of 600 mg/kg and 120 mg/kg, reporting better outcomes at 600 mg/kg regarding weight loss and colon length (Kawai et al., 2017). However, another study testing doses of 100, 200, and 400 mg/kg reported that 200 mg/kg IN exhibited better therapeutic efficacy than SASP (Liang et al., 2019). Based on our meta-analysis, the efficacy of doses above 600 mg/kg is generally confirmed, although definitive data remain limited. Additionally, the safety of a dosage of 1,000 mg/kg should be carefully considered. Future experiments should systematically determine the optimal dosage of IN before proceeding with further pharmacological evaluations.

### Research progress on the active ingredients of in IBD

These two effective monomers are believed to inhibit the expression of pro-inflammatory factors such as IL-6 and IL-1 β through the TRL4/MyD88/NF - κ B signaling pathway and upregulate the expression of AhR/CYP1A1 pathway, promoting the expression of anti-inflammatory factors such as IL-10 (Yang et al., 2021; Yang et al., 2023). However, there are currently no reports on the specific targets of these active ingredients in treating IBD. Then a network pharmacology prediction on the treatment of UC with IN indicated that the top five targets were TNF, PTGS2, JUN, NOS3, and PPARγ, and the highly associated pathways in the prevention and treatment of UC by QD include the IL-17 signaling pathway, T-cell receptor signaling pathway, VEGF signaling pathway, and Th1 and Th2 cell differentiation. Further research can be conducted in these areas.

### Strengths and limitations

This study provides evidence that supports the efficacy of IN for treating IBD in animal models and summarizes possible underlying mechanisms. Nevertheless, several limitations exist. First, the included studies were relatively few, and unpublished or recently published studies may have been overlooked, limiting the conclusions regarding the upper dosage limit of IN treatment. Additionally, current animal models primarily replicate general colitis. Due to differences in the pathological characteristics between CD and UC, these models may not fully reflect specific disease features. Thus, while IN can effectively ameliorate intestinal inflammation, its precise therapeutic role in specific disease subtypes remains uncertain. Although the study did not explicitly identify adverse reactions, it should be noted that the limited sample size and single model led to limitations in the conclusion.

## Conclusion

IN significantly reduced disease severity in animal models of IBD by suppressing inflammation through balancing innate and adaptive immunity, regulating the AhR/CYP1A1 signaling pathway, and modulating gut microbiota composition. Subsequent research should first clarify the optimal dosage and administration method of IN for treating IBD. Based on this, the main active ingredients of IN should be identified through mass spectrometry, and then the specific mechanism of action of IN’s active ingredients in treating IBD should be clarified through RNA sequencing, protein chips, or enzyme digestion methods.

## Data Availability

The original contributions presented in the study are included in the article/Supplementary Material; further inquiries can be directed to the corresponding author.

## Abbreviations

IN, indigo naturalis; IBD, bowel disease; UC, ulcerative colitis; CD, Crohn’s disease; 5-ASA, 5-aminosalicylic acid; DSS, dextran sodium sulfate; TNBS, trinitrobenzene sulfonic acid; OXZ, oxazolone; HE, hematoxylin–eosin; DAI, Disease Activity Index; IL, including interleukin; TNF-α, tumor necrosis factor-α; CAMARADES, Collaborative Approach to Meta Analysis and Review of Animal Data from Experimental Studies; MD, mean difference; MAMPs, microbe-associated molecular patterns; TLRs, toll-like receptors; RORγ, receptor-related orphan receptor γ; FOXP3, transcription factor forkhead box P3; AhR, aryl hydrocarbon receptor; CYP1A1, cytochrome P450 1A1; IL-10R1, IL-10 receptor; STAT3, signal transducer and activator of transcription 3; SCFAs, short-chain fatty acids.
